# Supplementary material for: Empirical methods for controlling false positives and estimating confidence in ChIP-Seq peaks
Source: BMC Bioinformatics. 2008 Dec 5;9:523. doi: 10.1186/1471-2105-9-523 (PMC2628906; doi:10.1186/1471-2105-9-523)
Supplement: Additional File 1 — A variety of html documents from the USeq web site detailing the available applications, their best usage, output file type descriptions, command line menus, etc. [file 1471-2105-9-523-S1.zip › USeqUserGuides/JavaDoc.zip!/JavaDoc/meme/MotifScanner.html]

MotifScanner


|  |  |  |
| --- | --- | --- |
| **Overview** \| **Package** \| **Class** \| **Use** \| **Tree** \| **Deprecated** \| **Index** \| **Help** | |  |
| **PREV CLASS**   **NEXT CLASS** | **FRAMES**    **NO FRAMES**     **All Classes** |  |
| SUMMARY: NESTED \| FIELD \| CONSTR \| METHOD | DETAIL: FIELD \| CONSTR \| METHOD |  |


---


## meme Class MotifScanner

```
java.lang.Object
  meme.MotifScanner
```

---

``` public class MotifScanner extends java.lang.Object ```

MotifScanner objects are used to scan sequences with a particular weight matrix and generate
an array of MotifHit objects.

---

**Constructor Summary**

`MotifScanner(double[][] logLikelihoodMatrix)`

`MotifScanner(double[][] logLikelihoodMatrix,
MemeResults res)`


| **Method Summary** | |
| --- | --- |
| `static int` | `calculateMaxCluster(MotifHit[] hits, int window)`             Calculates the maximum number of hits that occur within a given window size. |
| `double` | `findLowestScoringSeq(java.lang.String[] seqs)`             Returns the lowest score from scoring all the seqs. |
| `double[]` | `getBaseScores()` |
| `java.util.ArrayList` | `getMotifHits()` |
| `int` | `scanEm(java.lang.String[] seqs, java.lang.String[] names, double cutOff)`             Scans a set of sequences for the motif, saves results in a MemeResults object. |
| `void` | `scanPrintAllSequences(java.lang.String[] seqs, java.lang.String[] names)`             Scans a set of sequences for the motif, prints as it goes. |
| `void` | `scanPrintNumberHits(double scoreCutOff, java.lang.String[] seqs, java.lang.String[] names)`             Scans a set of sequences for the motif, prints as it goes. |
| `void` | `scanPrintSequences(java.lang.String[] seqs, java.lang.String[] names, double cutOff)`             Scans a set of sequences for the motif, prints as it goes. |
| `void` | `scanSequence(java.lang.String seq)`             Scans a sequence for the motif, scores every window, forward and reverse complement, assigning the higher score to the center base. |
| `MotifHit[]` | `scoreSequence(double cutOff, java.lang.String seq)`             Returns an array of MotifHits that are > or = to the loglikelihood cutOff. |
| `double` | `scoreSubSeq(java.lang.String seq)`             ignores non GATC chars |

**Methods inherited from class java.lang.Object**

`equals, getClass, hashCode, notify, notifyAll, toString, wait, wait, wait`

**Constructor Detail**

### MotifScanner

```
public MotifScanner(double[][] logLikelihoodMatrix,
                    MemeResults res)
```

---


### MotifScanner

```
public MotifScanner(double[][] logLikelihoodMatrix)
```


**Method Detail**

### getMotifHits

```
public java.util.ArrayList getMotifHits()
```

---


### calculateMaxCluster

```
public static int calculateMaxCluster(MotifHit[] hits,
                                      int window)
```

:   Calculates the maximum number of hits that occur within a given window size.
    Assumes that the array hasn't been sorted and is ordered by position.

---


### findLowestScoringSeq

```
public double findLowestScoringSeq(java.lang.String[] seqs)
```

:   Returns the lowest score from scoring all the seqs. Useful in getting a
    cutOff score when the seqs are the seqs used to generate the matrix.
    All the seqs must be the same size as the matrix!

---


### scanEm

```
public int scanEm(java.lang.String[] seqs,
                  java.lang.String[] names,
                  double cutOff)
```

:   Scans a set of sequences for the motif, saves results in a MemeResults object.

---


### scanPrintSequences

```
public void scanPrintSequences(java.lang.String[] seqs,
                               java.lang.String[] names,
                               double cutOff)
```

:   Scans a set of sequences for the motif, prints as it goes.

---


### scanPrintAllSequences

```
public void scanPrintAllSequences(java.lang.String[] seqs,
                                  java.lang.String[] names)
```

:   Scans a set of sequences for the motif, prints as it goes.

---


### scanPrintNumberHits

```
public void scanPrintNumberHits(double scoreCutOff,
                                java.lang.String[] seqs,
                                java.lang.String[] names)
```

:   Scans a set of sequences for the motif, prints as it goes.

---


### scoreSequence

```
public MotifHit[] scoreSequence(double cutOff,
                                java.lang.String seq)
```

:   Returns an array of MotifHits that are > or = to the loglikelihood cutOff.
    Set cutOff to a very negative number (ie -20\*lengthOfMotif) if you want all the MotifHits.
    Will not score any non GATC containing sequences. Searches both strands but returns coordinates
    for either in forward direction.
    Also assigning the higher score to the center base for every window (baseWindow double[]) where
    index 0 in the double[] is base 1 in the seq. 0s will be found at
    the beginning and end. Scores < 0 are assigned 0.

---


### scanSequence

```
public void scanSequence(java.lang.String seq)
```

:   Scans a sequence for the motif, scores every window, forward and reverse complement, assigning the
    higher score to the center base. Index 0 in the double[] is base 1 in the seq. 0s will be found at
    the beginning and end. Scores < 0 are assigned 0

---


### scoreSubSeq

```
public double scoreSubSeq(java.lang.String seq)
```

:   ignores non GATC chars

---


### getBaseScores

```
public double[] getBaseScores()
```


---


|  |  |  |
| --- | --- | --- |
| **Overview** \| **Package** \| **Class** \| **Use** \| **Tree** \| **Deprecated** \| **Index** \| **Help** | |  |
| **PREV CLASS**   **NEXT CLASS** | **FRAMES**    **NO FRAMES**     **All Classes** |  |
| SUMMARY: NESTED \| FIELD \| CONSTR \| METHOD | DETAIL: FIELD \| CONSTR \| METHOD |  |


---
